# A 598-bp InDel Variation in the Promoter Region of *Bna.SOC1.A05* Is Predominantly Present in Winter Type Rapeseeds

**DOI:** 10.3389/fpls.2021.640163

**Published:** 2021-04-13

**Authors:** Sarah Matar, Siegbert Melzer

**Affiliations:** Plant Breeding Institute, Christian-Albrechts-University of Kiel, Kiel, Germany

**Keywords:** *Brassica napus*, *Bna.SOC1*, flowering, structural variation, presence/absence variation

## Abstract

During rapeseed domestication and breeding, genetic diversity allowed to adapt it to different eco-geographical regions and to shape its useful traits. Structural variations (SVs), including presence/absence variations (PAVs), are thought to play a major role in the genetic diversity and phenotypic plasticity of rapeseed. In this study, we detected a 598-bp PAV within the promoter region of an *Arabidopsis* ortholog of a major flowering time gene and a downstream target of *FLC*, *SOC1*, which is one of the first genes that are upregulated in rapeseed during vernalization. Further analysis showed that the insertion is present predominantly in winter types while absent in spring types. The 589-bp sequence is present only in the A sub-genome indicating that it originated from *Brassica rapa*. Since the genomic region around *Bna.SOC1.A05* showed a strong reduction in nucleotide diversity, the insertion might represent a larger selected sweep for rapeseed adaptation. Cis-element analysis showed that the insertion contains an ACGTG box, which is the strongest binding motif for the HY5 transcription factor in *Arabidopsis*. In addition, expression analyses showed that mRNA levels of *Bna.SOC1.A05* were lower in accessions carrying the insertion compared to the ones that had no insertion.

## Introduction

Rapeseed (*Brassica napus*) is an allotetraploid crop formed by spontaneous interspecific hybridization of two diploid species; *Brassica rapa* and *Brassica oleracea* around 7,500 years ago (Chalhoub et al., 2014). In the last decades, rapeseed evolved rapidly to become one of the most important oil crops grown worldwide. Its dynamic allopolyploid genome plays a prime role in its diversification and adaptation to different natural environments. According to vernalization requirement, *B. napus* can be divided into three different growth types; winter, semi-winter, and spring rapeseed (Zou et al., 2019). Winter types are grown mainly in Europe; they have an obligate vernalization requirement, and are suggested to be the original type of rapeseed (Lu et al., 2019). Spring types are grown in Australia and North America and flower without any vernalization requirement. Semi-winter types were formed gradually after the introduction of winter types into China in the 20th century, where they were adapted to flower after a short period of vernalization (Qian et al., 2006). The divergence in growth types of rapeseed accessions has been associated mainly to allelic variation, represented by short (SNPs or InDels) or large-scale structural variations (SVs), within or upstream of flowering time genes, especially in vernalization responsive genes such as *Bna.FRI (FRIGIDA) Bna.FLC (FLOWERING LOCUS C)* and downstream targets like *Bna.FT* genes (*FLOWERING LOCUS T*; Wang et al., 2011; Wu et al., 2019).

Whole genome sequencing of *B. napus* revealed a highly complex and redundant genome, with high frequency of homologous exchanges between the two subgenomes (Chalhoub et al., 2014). Homologous exchanges can be translocations, duplications, and deletions, which lead to extensive presence/absence variations (PAVs) and segmental deletions, therefore representing an additional source of genetic diversity (Schiessl et al., 2017; Hurgobin et al., 2018). Several studies have revealed the association of PAVs with important agronomic traits such as disease resistance and oil content in rapeseed (Liu et al., 2012; Qian et al., 2016; Stein et al., 2017; Gabur et al., 2018). For instance, deletion of the orthologous *Arabidopsis* gene *NON-YELLOWING 1* (*NYE1*; *Bna.NYE.A01*) was associated with increased chlorophyll content, which simultaneously affected oil-content and adaptation to cooler environments (Qian et al., 2016). It is known that transposable elements are another important driving force for genomic diversity. They can affect gene functions through insertions into coding regions, or influence gene expression through insertion into regulatory elements or by affecting local chromatin structure (Lisch, 2013; Chuong et al., 2017; Anderson et al., 2019). For instance, the insertion of tourist-like miniature inverted-repeat transposable elements (MITE), upstream of the *Bna.FLC.A10* gene was found to be associated with vernalization requirement (Hou et al., 2012). Moreover, an 810-bp MITE insertion in exon 7 of *Bna.FLC.A02* silenced the gene and was found to be associated with a spring habit of rapeseed plants lacking the MITE insertion in *Bna.FLC.A10* (Yin et al., 2020). Recently, a study revealed that up to 10% of genes in the rapeseed genome are affected by SVs, for which half of these variations are between 100 to 1,000 bp long (Chawla et al., 2020). The study also reported a SV upstream of *Bna.FT.A02* that was associated with the eco-geographical distribution of different rapeseed cultivars. Therefore, studying SVs in flowering time genes might be a useful approach to further understand the history of evolution and adaptation of rapeseed and it might also facilitate the development of molecular markers to differentiate between different growth types.

Assuming that genomic variation in other flowering time genes might have also played a role in the evolution and divergence of rapeseed, we analyzed SVs within and upstream of orthologs of key downstream targets of *Bna.FLC* genes. We used the comprehensive resource of genomic sequences of a worldwide collection of 991 rapeseed germplasms (Wu et al., 2019) from which we selected 80 accessions with relatively high sequencing depth. As we compared the upstream regions of certain flowering time genes between spring and winter accessions, we uncovered a previously unidentified sequence insertion upstream of *Bna.SOC1.A05*, which is associated with winter types. We also confirmed this variation in the ASSYST *B. napus* diversity set (Bus et al., 2011). Further analysis suggested that the sequence was inserted in the promoter region of *Bna.SOC1.A05* after the merge of the two ancestral genomes. Evidence of a selective sweep was reflected by the dramatic reduction of nucleotide diversity in the region harboring this insertion compared to its surrounding region. The strong co-segregation of this SV with the crop type indicated an additionally and potentially crucial role of this genomic re-arrangement for the eco-geographical adaptation of rapeseed.

## Materials and Methods

### Plant Materials and Growth Conditions

For expression analysis, the two spring rapeseed accessions that carry the insertion, Bingo and Adamo, and the winter inbred line Express617, from ERANET-ASSYST panel (Bus et al., 2011), in addition to the spring accession Haydn that lacks the insertion, were used. Seeds were sown on soil in 9 cm pots and grown under greenhouse conditions in 16 h-light/8 h-dark cycles at 20°C. Flowering time was measured in days from sowing to opening of the first flower.

### RNA Extraction and Expression Analysis

For RT-qPCR, leaves and shoot apices were sampled at 2, 4, and 6 weeks after sowing. Apical buds were dissected under a binocular microscope using scalpel and forceps and immediately frozen in liquid nitrogen. Around 8–10 apical buds were pooled for one biological replicate. RNA isolation and DNase treatment were carried out according to the instruction manual provided with the PeqGold Total RNA Kit (PeqLab, Germany).

RT-qPCR was performed for three biological replicates each with three technical repeats for each time point. RT-qPCR was carried out using Platinum™ SYBR™ Green qPCR SuperMix (ThermoFischer Scientific). Expression levels were calculated with the comparative *Δ*ΔCt method (Livak and Schmittgen, 2001). Primers used for gene expression analysis are listed in (Supplementary Table S1). *Bna.ACTIN2* was used to normalize gene expression levels.

### Transcriptome Analysis

For RNA-Seq analyses, the raw RNA-Seq reads of 72 rapeseed accessions including 31 winter and 41 spring types from a previous transcriptomics study (Harper et al., 2012; Havlickova et al., 2018) were downloaded from SRA NCBI^1^ using parallel-fastq-dump. The reads were mapped to the Express617 genome (Matar et al., 2021) using STAR (Dobin et al., 2013; v.2.5.4). For transcriptome assembly, the RNA-Seq read alignments were used as an input file to run Cufflinks (v2.2.1; Trapnell et al., 2012). The Cufflinks module Cuffnorm was used to generate normalized transcript expression levels [normalized fragments per kilobase per million mapped reads (FPKM)] from all rapeseed accessions studied. Expression levels of six *Bna.SOC1* genes were plotted in RStudio (Boston, Massachusetts), using the data visualization package ggplot2 (Wickham, 2011).

### Sequence Analysis and Detection of Presence/Absence Variation

Whole genome sequencing data of 80 rapeseed accessions were downloaded from SRA NCBI (https://www.ncbi.nlm.nih.gov/sra/SRP155312; Wu et al., 2019) using parallel-fastq-dump. Reads of each accession were mapped to the Darmor-*bzh*-based genome assembly of winter rapeseed Express617 (Matar et al., 2021) using Burrows-Wheeler Aligner mem (BWA-MEM v.0.7.17) software package (Li and Durbin, 2009). Visual detection of read alignments was performed using the Integrative Genome Viewer (IGV). Per base depth of coverage was calculated for each accession at the genomic region of six *Bna*.*SOC1* orthologs in the Express617 genome, including 3,000 bp upstream, using Samtools (Li et al., 2009). PCR using genomic DNA from the ASSYST *B. napus* diversity set of different spring and winter rapeseed accessions (Bus et al., 2011), followed by Sanger sequencing was used to further confirm the PAV. Sequence alignment was visualized on the multiple sequence alignment visualization and editing program Jalview (Waterhouse et al., 2009).

NSITE-PL (Solovyev et al., 2010) was used to analyze the putative promoter region and the composition of functional motifs upstream of *Bna.SOC1.A05*, of other *Bna*.*SOC1* orthologs in addition to *At.SOC1*.

### Phylogenetic Analysis

For evolutionary analyses, SNPs within 10.5 kb genomic fragments including the insertion and 5,000 bp flanking sequence were extracted from whole genome SNP files of 80 rapeseed accessions (Wu et al., 2019). The neighbor-joining tree cladogram was constructed using Tassel software package (v5.2.59), and visualized using iTOL. To study whether the upstream region of *Bna.SOC1.A05* was under selection, nucleotide diversity (*π*) was calculated for chromosome A05 using VCFtools (v0.1.13) in a 1,000 bp sliding window with 100 bp steps.

### Screening of Homologous Sequences in *Brassica napus* and *Brassica rapa* Genomes

To identify homologous sequences, the inserted sequence was queried against the Express617 and *B. rapa* (Chiifu-401-42) reference genomes using BLAST+ package from NCBI. Circos software package (Krzywinski et al., 2009) was used to visualize the position of the insertion sequence in *B. napus* and *B. rapa* genomes. RepeatMasker Web Server^2^ and MITE-Tracker (Crescente et al., 2018) were used to search for putative repetitive and transposable elements. Multiple sequence alignments and phylogenetic analyses of homologous sequences from *B. napus* and *B. rapa* were performed using the multiple sequence alignment program Clustal Omega (McWilliam et al., 2013).

## Results

### A 598-bp PAV Upstream of *Bna.SOC1.A05* Co-segregates With Crop Type in Rapeseed

**Figure 1:**
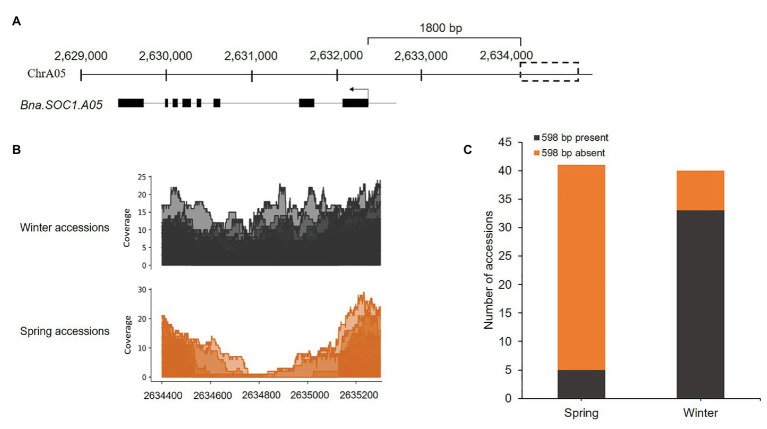
Presence/absence variation (PAV) upstream of *Bna.SOC1.A05* is associated with growth type. **(A)** Genomic region harboring *Bna.SOC1.A05* and the PAV in Express617 genome (dotted box). **(B)** Per base depth of coverage in the region 2,634,400 to 2,635,200, showing drop in coverage in the spring accessions. **(C)** Stack plot showing the presence of the insertion in 85% of the winter accession and deletion in 87% of the spring accessions studied.

Previously, we identified certain flowering time genes that were upregulated in the shoot apical meristem in winter rapeseed upon the downregulation of *Bna.FLC* during vernalization. These genes included orthologs of the flowering time integrator and downstream target of *FLC*, *SOC1*, in addition to certain members of the *SPL* gene family (Matar et al., 2021). To study SVs between spring and winter rapeseed cultivars, within or upstream of the identified genes, we used the whole genome sequencing reads of 40 winter and 40 spring accessions, with an average sequencing depth ranging from 5 to 23. The reads were aligned to our Darmor-bzh reference-based genome assembly of the winter rapeseed cultivar Express617 (Matar et al., 2021), using Bwa-mem (v.0.7.17). We first carried out a visual detection of read alignments using IGV with a mapping quality threshold of 5. We observed that certain accessions had no reads that mapped upstream to a distinct region of *Bna.SOC1.A05* (Supplementary Figure S1) indicating the presence of a SV upstream of the gene. A closer examination showed that most of the spring accessions had no reads mapping to this region, while in most of the winter accessions reads were mapping. To further ascertain the boundaries of this SV, we used Samtools to calculate the depth of coverage in the genomic regions including the gene sequence and 3,000-bp upstream of *Bna.SOC1.A05*. At 1.8-kb upstream of *Bna.SOC1.A05* (Figure 1A), the sequencing coverage in spring accessions gradually dropped to zero, while in winter accessions it remained at the same level (Figure 1B). Average sequence coverage was higher in winter types compared to spring types because certain spring types had low sequencing depth (Figure 1B). The sequence-depth analysis further confirmed the segregation of the SV between winter and spring types, where more than 87% of the spring accessions showed absence of the genomic sequence at the analyzed region, while 85% of the winter accessions showed the presence of the insertion (Figure 1C, Supplementary Table S2). Therefore, we concluded that this SV upstream of *Bna.SOC1.A05* segregates with the growth types of *B. napus*.

None of the other analyzed *Bna.SOC1* paralogs or the *Bna.SPL* genes showed a SV similar to that observed for *Bna.SOC1.A05*. Based on SNP data of a 10.5-kb region flanking the insertion, we performed a phylogenetic analysis and found that the 80 rapeseed accessions were clustered into two groups that corresponded largely to winter and spring crop types, which suggested that the genomic variation in the region containing the insertion and *Bna.SOC1.A05* might have contributed to growth-type diversification (Supplementary Figure S3).

### The 598-bp Insertion in the Upstream Region of *Bna.SOC1.A05* Originated From *Brassica rapa*

Low sequencing depth is not always reliable to detect SVs; therefore, we further validated the PAV upstream of *Bna.SOC1.A05 via* PCR with flanking primers and using genomic DNA from spring and winter accessions of the ASSYST-PANEL (Bus et al., 2011). The variation could be clearly distinguished by PCR, and showed that an insertion is present in 25 out of 30 of the winter types but absent in 27 out of 30 of the spring types (Supplementary Table S3). Out of the 60 tested accessions from the ASSYST panel, 22 accessions were common with accessions from the whole genome sequencing data. The PCR results of the 22 rapeseed accessions were in accordance with the whole genome sequencing data.

**Figure 2:**
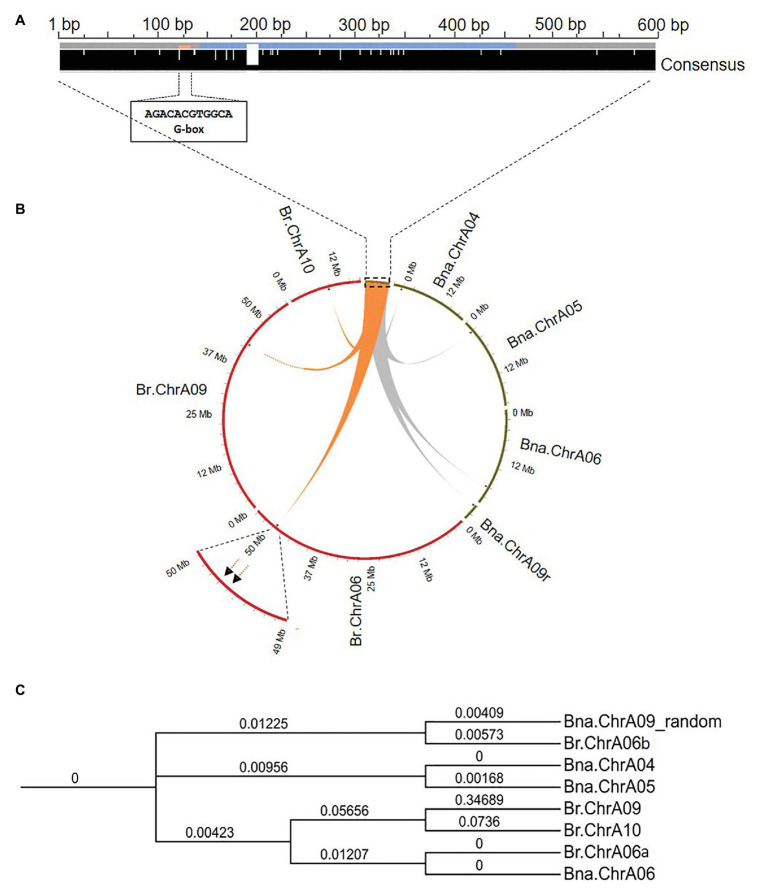
Sequence analysis and genomic positions of the insertion-homologous sequences in *Brassica napus* and *Brassica rapa*. **(A)** Multiple sequence alignment of the four homologous sequences from *B. napus*, showing consensus (black bar) with a high modal residue per column, and the postion of the G-box HY5 binding motif. Blue color in the middle of the insertion sequence corresponds to the region matching to *SRO2*. **(B)** Circos plot showing genomic positions of the insertion-homologous sequences on chromosomes of *B. rapa* (red) and *B. napus* (green). **(C)** Phylogenetic tree showing the evolutionary relationship between the eight identified sequences in the genomes of *Bna. napus* and *B. rapa*.

The PCR products from different rapeseed genotypes were Sanger sequenced and aligned. The insertion upstream of *Bna.SOC1.A05* and the region flanking the insertion site showed a high sequence identity in all accessions tested (98–100%), indicating that this intergenic region is conserved among different rapeseed genotypes (Supplementary Figure S2). The insertion sequence had no sequence similarity to repetitive and transposable elements on the RepeatMasker web server. In addition, the MITE annotating software, MITE Tracker (Crescente et al., 2018) did not annotate the insertion sequence as a putative MITE. Further sequence analysis showed that the first 151 nucleotides are only present in these sequences, however, the following 313 nucleotides, showed a high sequence identity (*e*-value < 7*e*-29) with the gene *SIMILAR TO RCD ONE 2* (*SRO2*) from *Arabidopsis*. SRO2 proteins have a poly (ADP-ribose) polymerase (PARP) domain, which is a signature domain known to modify a variety of proteins by attaching the ADP-ribose-moiety from NAD+ to the target molecule (Kraus and Lis, 2003). PARPs are best studied in mammals and they have a broad range of functions, including DNA repair, genome integrity, and epigenetic regulation (Citarelli et al., 2010). Analysis of cis-regulatory elements in the inserted fragment uncovered an ACGTG box, which has been shown to be the strongest element for binding the ELONGATED HYPOCOTYL 5 (HY5) transcription factor in *Arabidopsis* (Burko et al., 2020; Figure 2A). It is worth mentioning that four functional homologs of the *SRO2* gene are present with two copies in each subgenome of rapeseed.

To trace the origin of the 598-bp DNA fragment, we performed a local blast search and identified three homologous sequences of the insertion sequence (*e*-value = 0.0) that were present only in the A subgenome (Figure 2B; Supplementary Table S4), indicating that the sequence might have originated from *B. rapa*. A local blast search of the insertion sequence identified four homologous sequences (*e*-value < 9.41*e*-75) in the *B. rapa* genome (Chiifu-401-42; Supplementary Table S5). However, none of the homologous sequences that were identified in *B. rapa*, were located on chromosome A05 (Figure 2B), and the upstream region of *Br.SOC1.A05* was almost 100% identical to the upstream sequence of *Bna.SOC1.A05* in the spring rapeseed accessions. Chromosome A06 in *B. rapa* carries two copies of the insertion, while the other copies are present on chromosomes A10 and A09 (Figure 2B). We also analyzed sequences of other *B. rapa* accessions, including winter turnip that was suggested to be the ancestor of *B. napus* (Lu et al., 2019), but no insertion was found on chromosome A05. This indicates that, although the sequence was present in the *B. rapa* genome, it was inserted upstream of *Bna.SOC1.A05* only after the hybridization of the two ancestral genomes.

To estimate the evolutionary relationship between the identified homologous sequences from *B. napus* and *B. rapa* genomes, we conducted a phylogenetic analysis using multiple sequence alignment of the eight identified sequences. Phylogenetic analysis of the homologous sequences was constructed using Clustal Omega and resulted in four distinct pairs (Figure 2C), where the sequence on chromosome A06 in *B. rapa* (Br.ChrA06a) and its surrounding sequences showed 100% identity to that on Bna.ChrA06, indicating that the sequence on Bna.ChrA06 is the same as that on Br.ChrA06. The other sequence on the *B. rapa* chromosome A06 (Br.ChrA06b) showed a high sequence identity to the insertion sequence on Bna.ChrA09-random (Figure 2C). Insertions on chromosomes A04 and A05 in *B. napus* formed a separate clade, and showed less sequence identity to sequences on Br.ChrA10 and Br.ChrA09 compared to that on Br.ChrA06 (Figure 2C; Supplementary Table S5), suggesting that the sequences on Bna.ChrA04 and Bna.ChrA05 are distinct from the sequences on Br.ChA10 and Br.Chr.A09, and that they might have arisen in the *B. napus* genome and were not present the *Br. rapa* genome.

### Reduced Nucleotide Diversity in the Genomic Region Surrounding *Bna.SOC1.A05*

**Figure 3:**
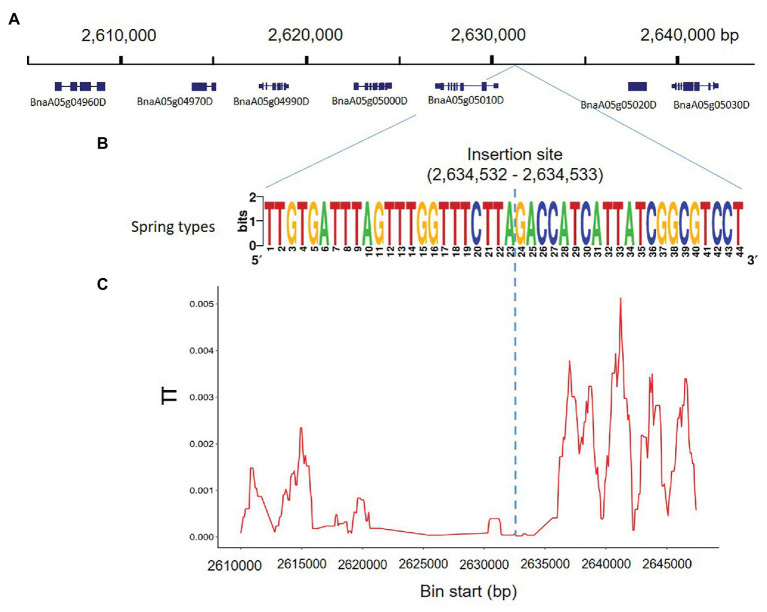
Nucleotide diversity accross the insertion site on chromosome 5. **(A)** 0.4-Mb genomic region surrounding the insertion sequence, and the genes annotated in this region according to Darmor-*bzh* reference genome. **(B)** The consenus sequence covering the breakpoint of the PAV in 18 rapeseed accessions. The breakpoint of the PAV is located between 2,634,532 and 2,634,533 in Darmor-*bzh* reference genome. **(C)** Nucleotide diversity (*π*) calculated based on SNP data of the 80 studied rapeseed accessions showing reduced nucleotide diversity in the region 2,620,000–2,635,000 surrounding the PAV.

To determine whether the genomic region harboring the insertion was potentially affected by selection, we used whole genome SNP information, of the 80 rapeseed accessions studied, to analyze nucleotide diversity in the region around *Bna.SOC1.A05* (*BnaA05g05010D*), compared to the nucleotide diversity of 40-kb surrounding that region (Figure 3A). The rapeseed accessions exhibited dramatically reduced nucleotide diversity in a genomic region of 15 kb (Figures 3B,C). In addition to *Bna.SOC1.A05*, a homolog of the MADS-box gene *AGAMOUS-LIKE 6* (*AGL6*; *BnaA05g05000D*) is also present in this region (Figure 3A). The low level of diversity in the genomic region suggests that the region has evolved under functional constraints.

### *Bna.SOC1.A05* Is Highly Expressed in Spring Types Lacking the Insertion

**Figure 4:**
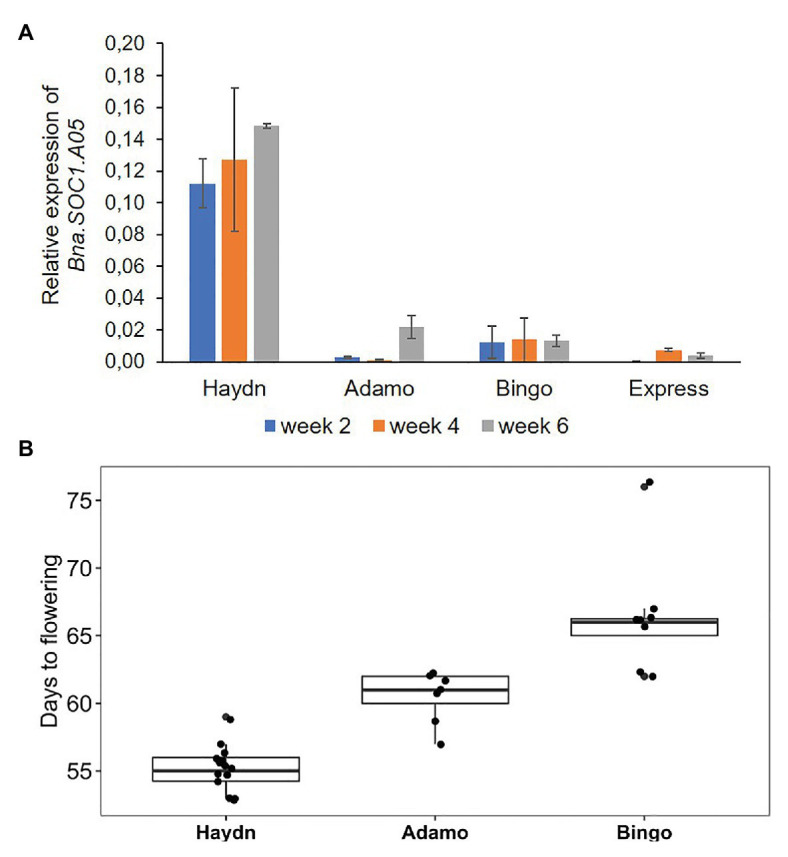
Expression and flowering time variation in different rapeseed accessions. **(A)** Expression levels of *Bna.SOC1.A05* in the apices four different rapeseed accessions, 2, 4, and 6 weeks after sowing. Expression was normalized to *Bna.Actin2*. Error bars represent SEM of three biological replicates. **(B)** Days to flowering determined by scoring the opening of the first flower in three different spring accessions.

To get an idea whether the PAV is associated with transcriptional activity of the *Bna.SOC1.A05* gene, we compared the expression of three spring accessions and one winter rapeseed accession. Two spring accessions that carried the insertion upstream of *Bna.SOC1.A05* were from the ERRANET-ASSYST panel, and the third spring accession was Haydn that has no insertion. The winter accession was Express617 that carries the insertion. We analyzed expression levels in apices of 2, 4, and 8 week-old plants and found that the expression of *Bna.SOC1.A05* was consistently lower in Bingo and Adamo compared to Haydn (Figure 4A). In fact, the expression levels of *Bna.SOC1.A05* were more comparable to that of the winter accessions Express617 (Figure 4A). In addition, Haydn flowered around 55 days after sowing, while Adamo and Bingo flowered 5 and 10 days later (Figure 4B). However, the expression of *Bna.SOC1.A05* did not explain the difference in flowering time between Bingo and Adamo, as Adamo was earlier flowering, but exhibited lower expression levels of *Bna.SOC1.A05*.

To further study the expression level of *Bna.SOC1.A05* in comparison to other *Bna.SOC1* genes, we analyzed the leaf transcriptome of 72 rapeseed accessions, including 31 winter and 41 spring accessions. The whole transcriptome reads (Havlickova et al., 2018) were downloaded from SRA NCBI and mapped to the Express617 genome assembly. The 60 ASSYST panel accessions that were genotyped for the PAV were also included in the RNA-seq analysis.

**Figure 5:**
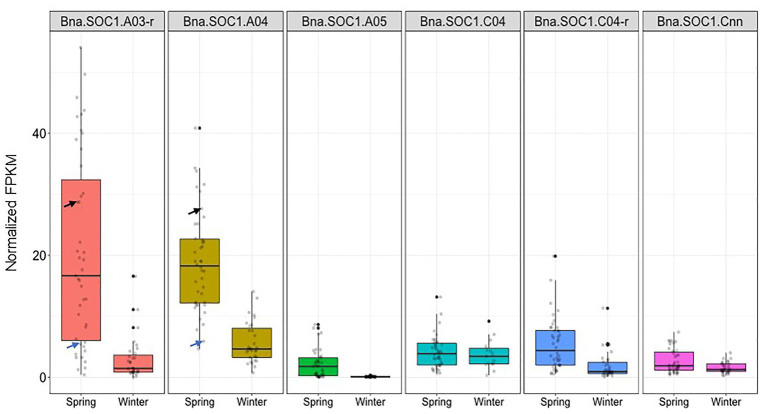
Expression levels of *Bna.SOC1* genes in spring and winter rapeseed accessions. Boxplot of normalized fragments per kilobase per million mapped reads (FPKM) values of the six *Bna.SOC1* paralogs in leaf transcriptome of 31 winter and 41 spring rapeseed accessions. Black arrow corresponds to the normalized FPKM values of Adamo, while blue arrows correspond to the normalized FPKM values of Bingo.

The expression levels of all *Bna.SOC1* genes in winter accessions were significantly lower than the expression of *Bna.SOC1* in spring accessions, except for *Bna.SOC1.C04* (Figure 5). This is expected considering that flowering in winter types is vernalization dependent, and that winter types are expected to have higher levels of *Bna.FLC* (Schiessl et al., 2019). In spring types, *Bna.SOC1.A03-random* and *Bna.SOC1.A04* showed the highest expression levels compared to other *Bna.SOC1* genes (Figure 5). Interestingly, in winter types, *Bna.SOC1.A05* was the only gene that was not detected before vernalization (Figure 5). In a transcriptome analysis of the winter accession during the course of vernalization, *Bna.SOC1.A05* was the first gene to be induced during floral transition (Matar et al., 2021). This tight vernalization-controlled expression of *Bna.SOC1.A05* further reinforces a pivotal role of this gene for the vernalization-driven floral transition in winter rapeseed. Further analysis of *Bna.SOC1* expression showed that the expressions levels *Bna.A03-random* and *Bna.SOC1.A04* were higher in Adamo compared to Bingo (Figure 5), which might explain the earlier flowering of Adamo.

## Discussion

In addition to SNPs and InDels, SVs, represented mainly by PAVs are prevalent in the *B. napus* genome, and are believed to be a driving force for its diversification and adaptation to different environments. Several studies have investigated the genetic variation of flowering-time divergence among the three rapeseed crop types. Most of the studies focused on the orthologs of *FLC*, as major genes contributing to the vernalization requirement, thus to the divergence between spring and winter types. PAVs upstream of certain *Bna.FLC* genes have been shown to be associated with flowering-time divergence in rapeseed (Hou et al., 2012; Yin et al., 2020).

Since downstream targets of *Bna*.*FLC* genes, might have also played a role in the evolution and diversification of flowering-time in rapeseed, we analyzed SVs within and upstream of orthologs of key downstream targets of *Bna*.*FLC*. Utilizing the comprehensive resource of genomic sequences of a worldwide collection of 991 rapeseed accessions (Wu et al., 2019), we looked for SVs within or upstream of orthologs of *Bna.SOC1* genes. Detection of SVs requires a high sequencing depth; therefore, we started to analyze only accessions that had a sequencing depth of 10X or more. However, out of 991 accessions, 188 were spring types, from which only few had sequencing depths of more than 10X (Wu et al., 2019). Therefore, our set of spring types included accessions with sequencing depth of 5 and 6X.

*SOC1* is a main floral integrator and a downstream target of *FLC* in *Arabidopsis* (Searle et al., 2006; Lee and Lee, 2010). In a previous study, we showed that two *Bna.SOC1* paralogs, *Bna.SOC1.A05* and *Bna.SOC1.C04-random*, are significantly induced during vernalization (Matar et al., 2021), suggesting a significant role of these two genes in floral transition. Analysis of the upstream regions of the *Bna.SOC1* genes, uncovered a 598-bp PAV upstream of *Bna.SOC1.A05*. The PAV is located 1.8-kb upstream of the transcription start site, suggesting that it is part of the promoter region of *Bna.SOC1.A05*. We used genomic DNA of accessions from the ERANET-ASSYST panel to further confirm the PAV by PCR. More than 90% of the tested spring accessions lacked the insertion, while around 84% of the winter accessions had the insertion (Supplementary Table S3), indicating that this PAV is associated with crop type in rapeseed.

Although it is in an intergenic region, sequencing of the insertion from different rapeseed accessions showed that the sequence is highly conserved, which suggested that the insertion is of functional importance and might contain certain motifs that are relevant to the activity of the downstream gene. Motif analysis revealed a CACGTG box, which is known to be a binding site for the transcription factors HY5 (Burko et al., 2020). In *Arabidopsis*, *SOC1* mediates the crosstalk between cold response and flowering through regulating cold-responsive (COR) genes (Seo et al., 2009). *SOC1* is known to be a downstream target of HY5, but through the binding motif CACGTA (Lee et al., 2007), which was also present in the promoter region of *Bna.SOC.A05*. Using SNP data from Wu et al. (2019), we observed that the sequence conservation is not only in the inserted sequence but also is extended to the surrounding genomic region. This extremely low nucleotide diversity was probably caused by a selective sweep including the presence of the insertion in winter types while its absence in spring types.

Presence/absence variations in the promoter regions can affect the expression level or pattern of the corresponding downstream gene. Changes in expression might be due to new cis-regulatory elements that are introduced to the region, or due to the elimination of certain motifs that were present at the breakpoint of the insertion. Changes in expression could also be a result of DNA methylation levels in the inserted sequence. In a study that aimed to understand the evolution of *SOC1* sequences, expression and regulation in *Brassica* species, Sri et al. (2020) showed that divergence in regulatory sequences was correlated with the expression divergence of different *SOC1* orthologs in *Brassica juncea*. The study also showed that *Brassica SOC1* promoters comprised most of *A. thaliana* transcription factor binding sites (TFBSs), with homolog specific loss or conservation of certain binding sites. The effect of loss of certain TFBS was tested *via* analysis of reporter gene expression pattern. For instance, one of the *SOC1* homologs (*BjuSOC1AAMF1*) had lost its function due to loss of critical TFBS in its promoter region. In addition, Sri et al. (2020) analyzed sequence length and distribution of TFBS in promoters of six *SOC1* homologs in *B. napus* which also showed diversification in length and TFBS content. In our analysis, the expression level of *Bna.SOC1.A05* in the two accessions that contained the insertion was significantly lower than in the spring accessions that lacked the insertion. Since the expression levels of *Bna.SOC1.A05* did not correlate with flowering time, we analyzed the expression levels of other *Bna.SOC1* paralogs using leaf transcriptome data from Havlickova et al. (2018). Interestingly, *Bna.SOC1.A05* was the only gene that was not expressed in 2-week-old winter type plants, indicating that it might be involved in the vernalization driven flowering response in winter rapeseed. In spring types, *Bna.SOC1.A03* showed the highest expression levels, with a higher level in Adamo compared to Bingo, which is in line with the observed flowering time in these two varieties. However, these transcriptome data were from 2-week-old plants and might not reflect the activity of the genes directly before and during the transition to flowering.

Further analyses revealed that the inserted sequence has three homologous sequences in the *B. napus* A genome, indicating that the sequences originated from the *B. rapa* ancestor. Surprisingly, analyses of homologous sequences in *B. rapa* showed that the sequences are not located on the same chromosomes as in *B. napus* A genome, except for one sequence that is present on chromosome A06, indicating that these sequences might be mobile by a hitherto unknown mechanism. As the upstream region of *Br.SOC1.A05* was similar to that in spring types, it is evident that this 598-bp sequence was introduced to the upstream region of *Bna.SOC1.A05* after the hybridization event. The known PAVs upstream of certain *Bna.FLC* genes are MITE-like transposable elements that were activated after the generation of *B. napus* and got introduced to the upstream regions of *Bna.FLC* also after hybridization (Hou et al., 2012). However, as our analyses showed that the 598-bp DNA sequence upstream of *Bna.SOC1.A05* is not a transposable element, we were further intrigued to determine how the new insertion appeared on different chromosomes in the *B. napus* genome. The phylogenetic analysis showed that the sequences on chromosomes Bna.ChrA04 and Bna.ChrA05 are distinct from the ones that were found on chromosomes A09 and A10 of *B. rapa* suggesting that they originated in the *B. napus* genome. Although the insertion sequence seems to include a fragment of a PARP gene, the fact that it contains two other unique sequences that are surrounding the PARP fragment and are conserved in all the insertion sequences found in *B. napus*, it is conceivable that the origin of these new sequences is more likely to be from a homologous sequence which was already present in the genome such as that on chromosome A06 and not from any of the SRO2 genes present in the genome. The mechanism by which the exact size of the sequence got translocated or multiplied in the genome is not understandable by current known transposition mechanisms. However, the strong association of this PAV with growth types and the association with *Bna.SOC1.A05* expression might either indicate that this PAV is of functional relevance or was transmitted to all current winter types through breeding history.

## Data Availability Statement

The original contributions presented in the study are included in the article/Supplementary Material, further inquiries can be directed to the corresponding author.

## Author Contributions

SMa designed and performed the experiments, conducted the data analysis, and wrote the manuscript. SMe designed the experiments and wrote the manuscript. All authors contributed to the article and approved the submitted version.

### Conflict of Interest

The authors declare that the research was conducted in the absence of any commercial or financial relationships that could be construed as a potential conflict of interest.

## Footnotes

^1^https://www.ncbi.nlm.nih.gov/search/all/?term=PRJNA309367

^2^http://www.repeatmasker.org
