# Supplementary material for: A 598-bp InDel Variation in the Promoter Region of Bna.SOC1.A05 Is Predominantly Present in Winter Type Rapeseeds
Source: Front Plant Sci. 2021 Apr 13;12:640163. doi: 10.3389/fpls.2021.640163 (PMC8078208; doi:10.3389/fpls.2021.640163)
Supplement: Supplementary file 1 [file Data_Sheet_1.docx]

Supplementary Material

# Supplementary Figures and Tables

## Supplementary Figures

Supplementary Figure 1: Screenshot from Integrative Genomics Viewer (IGV) showing read alignment results in a 7-kb region (track 1, top) within and upstream of *Bna.SOC1.A05* (track 17, bottom). A: Sequence coverages of 15 spring accessions mapped to Express617. B: Sequence coverages of 15 winter accessions mapped to Express617.

**Supplementary Figure 2:** Multiple sequence alignment of the PAV region in 20 rapeseed accessions showing high sequence conservation in the insertion and the region surrounding it. The insertion present in the winter accessions is marked in red. The black bar corresponds to the consensus sequence with a high modal residue per column indicating the conservation of the sequence between the different accessions.

**Supplementary Figure 3:** Phylogenetic Neighbor-joining tree. The tree was constructed based on SNP data in a 10.5-kb genomic region surrounding the insertions, using Tassel and plotted using iTOL. Green color represents winter accessions and orange color represents spring accessions. Red branch represents accessions without the insertion while blue branch represents accessions with insertion.

## Supplementary tables

**Supplementary Table 1:** List of primers used in this study.

**Supplementary Table 2:** List of 80 winter and spring accessions with whole genome sequencing data that were analyzed for the presence absence variation upstream of Bna.SOC1

**Supplementary Table 3:** List of accessions from ERANET ASSYST *B. napus* diversity set tested by PCR for the presence or absence of the insertion.

**Supplementary Table 4:** Results of local blast of the insertion sequence upstream of *Bna.SOC1.A05* against Express617 genome.

**Supplementary Table 5:** Results of local blast of the insertion sequence upstream of *Bna.SOC1.A05* against *B. rapa* Z1 genome.

**Supplementary Figure 1**: Screenshot from Integrative Genomics Viewer (IGV) showing read alignment results in a 7-kb region (track 1, top) within and upstream of *Bna.SOC1.A05* (track 17, bottom). A: Sequence coverages of 15 spring accessions mapped to Express617. B: Sequence coverages of 15 winter accessions mapped to Express617.

B

A


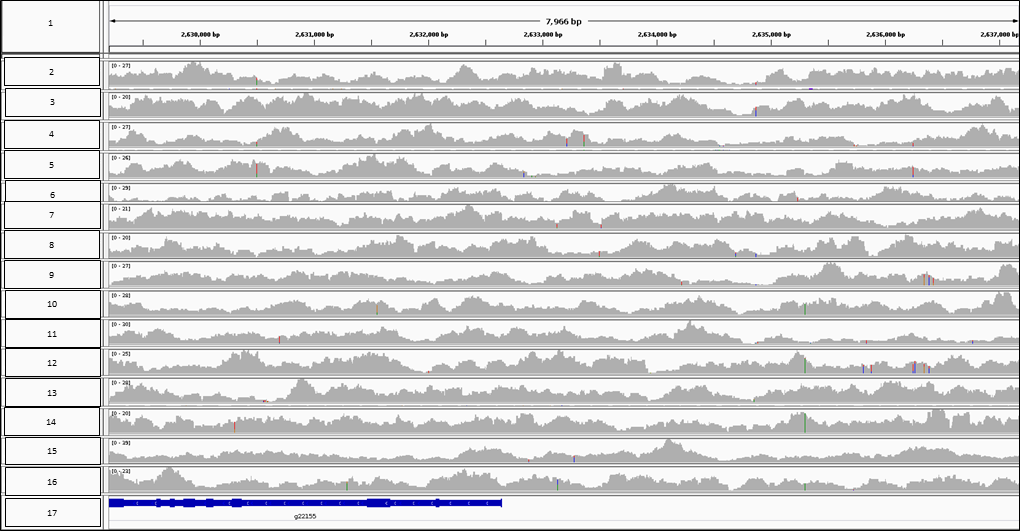

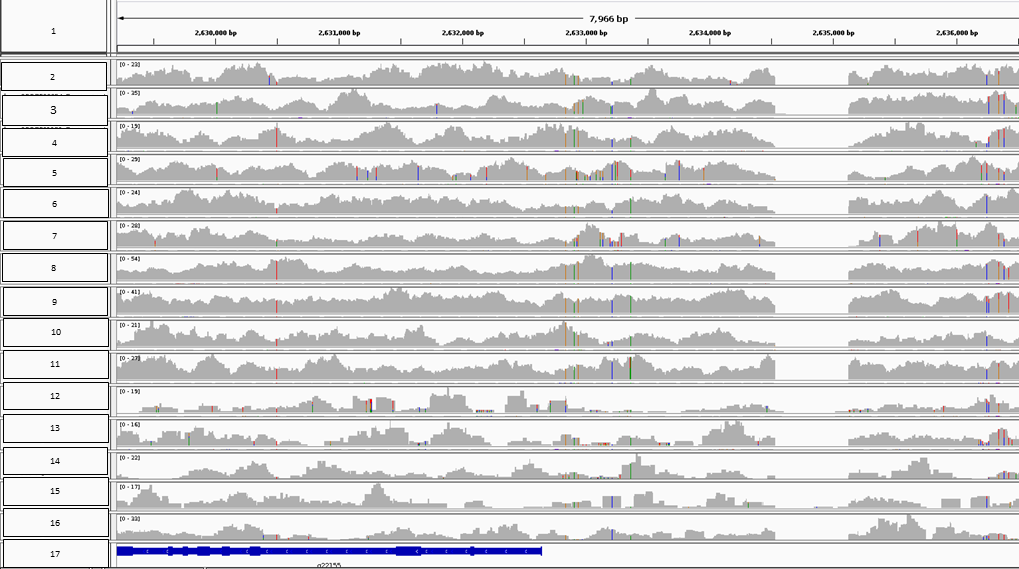


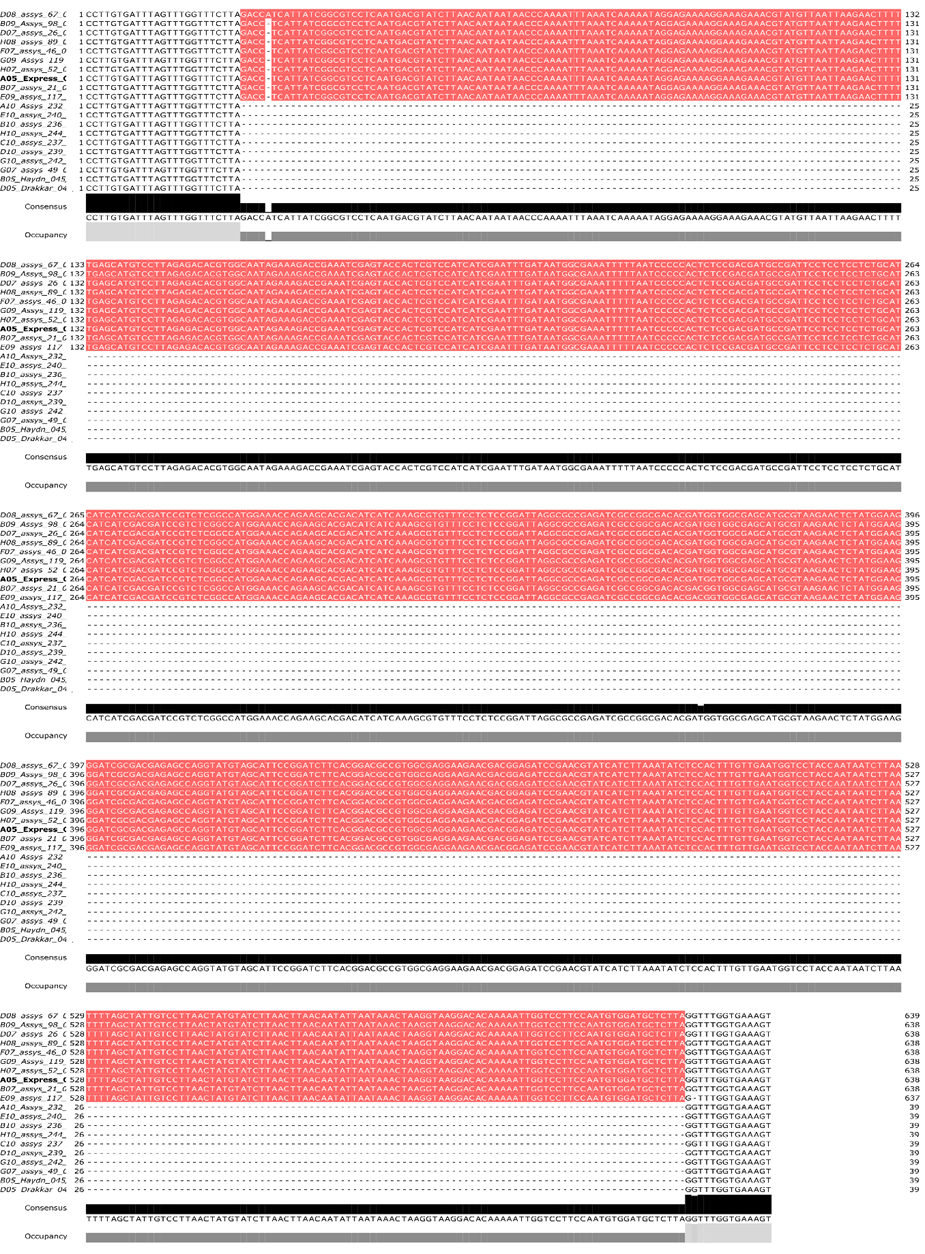


**Supplementary Figure 2**: Multiple sequence alignment of the PAV region in 20 rapeseed accessions showing high sequence conservation in the insertion and the region surrounding it. The insertion present in the winter accessions is marked in red. The black bar corresponds to the consensus sequence with a high modal residue per column indicating the conservation of the sequence between the different accessions.


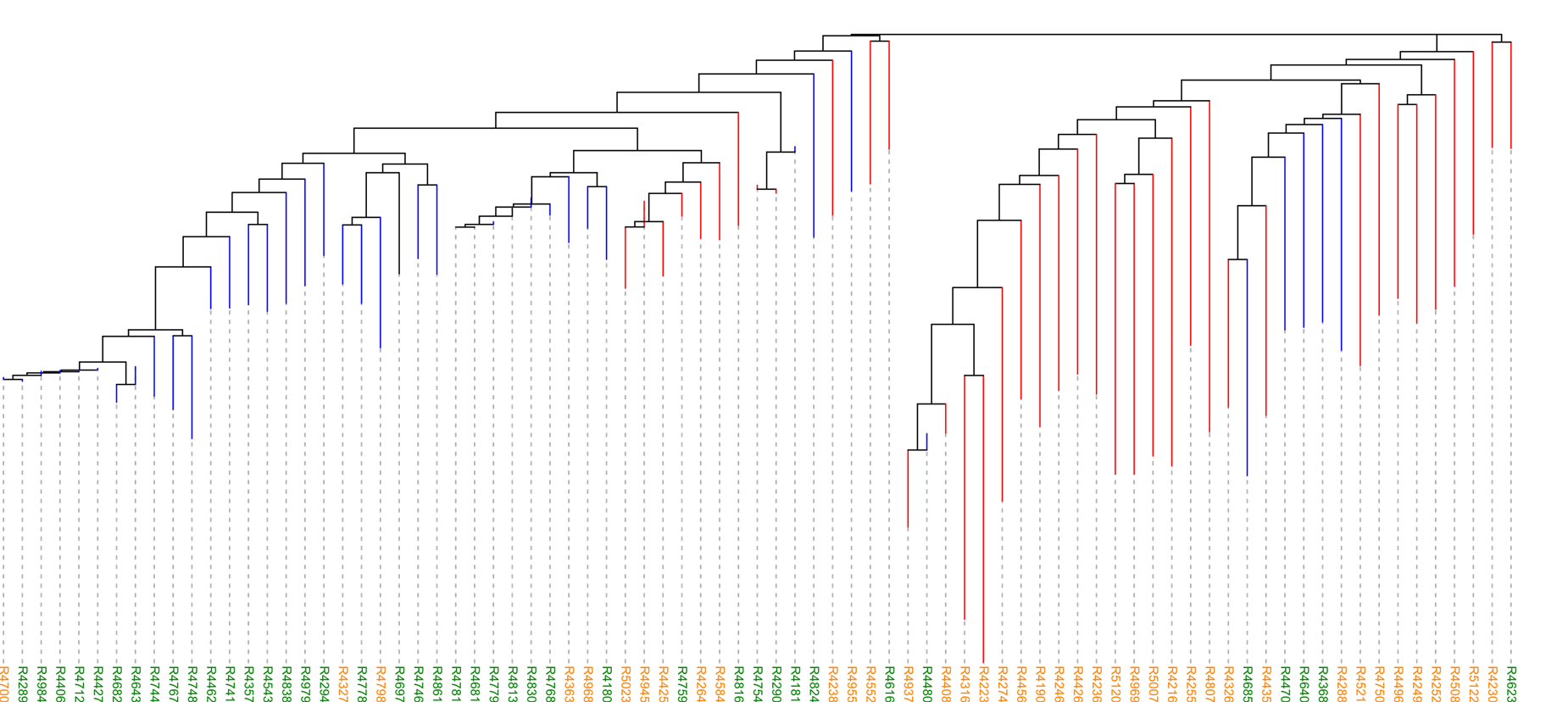


**Supplementary Figure 3**: Phylogenetic neighbor-joining tree. The tree was constructed based on SNP data in a 10.5-kb genomic region surrounding the insertions, using Tassel and plotted using iTOL. Green color represents winter accessions and orange color represents spring accessions. Red branches represent accessions without the insertion while blue branches represent accessions with insertion.

Supplementary Table 1: List of primers used in this study

| Primer name | Primer sequence 5’-3’ | Amplicon | |
| --- | --- | --- | --- |
| SM95_fwd | GTCCAGAAACACTCCGTTCG | Insertion | |
| SM95_rev | AATCACCAAACCAACGAGATG |  |  |
| SM100_fwd | CTATAGTTTTGTTTTGAAAGGGAAAC | *Bna.SOC1.A05* |  |
| SM100_rev | AGAAACTTCAGCATCACAAAGA |  |  |
| Act_1 | TCTGGTGATGGTGTGTCTCA | *Bna.Actin2* |  |
| Act_1 | GGTGAACATGTACCCTCTCTCG |  |  |

Supplementary Table 2: List of 80 winter and spring accessions with whole genome sequencing data that were analyzed for the presence absence variation upstream of *Bna.SOC1*

| Accession No. | Name | Origin | Growth type | Sequenced depth | SRR_id | insertion |
| --- | --- | --- | --- | --- | --- | --- |
| 26 | **Sl 524** | Czechoslovakia | winter | 10 | SRR7601059 | yes |
| 147 | **Darmor** | France | winter | 10 | SRR7601146 | Yes |
| 148 | **Bienvenue, R 33** | France | winter | 10 | SRR7601145 | No |
| 152 | **Titus** | France | winter | 9 | SRR7601152 | Yes |
| 301 | **Planet** | Germany | winter | 12 | SRR7601120 | Yes |
| 387 | **Wrg 15** | Germany | winter | 9 | SRR7600976 | Yes |
| 397 | **Hermes** | Germany | winter | 9 | SRR7601107 | Yes |
| 487 | **Gesunder** | Germany | winter | 10 | SRR7601073 | Yes |
| 565 | **Bnw 1.82/86** | GDR | winter | 9 | SRR700963 | No |
| 572 | **SR 413** | GDR | winter | 12 | SRR7600968 | No |
| 590 | **Bnw 1.63/83** | GDR | winter | 9 | SRR7600866 | Yes |
| 593 | **Bnw 9** | GDR | winter | 10 | SRR7600896 | Yes |
| 631 | **SR 417** | GDR | winter | 9 | SRR7631348 | Yes |
| 632 | **Bnw 18** | GDR | winter | 9 | SRR7631349 | Yes |
| 636 | **Ir 1** | Hungary | winter | 13 | SRR7631344 | Yes |
| 648 | **Sinapri** | Italy | winter | 9 | SRR7631222 | Yes |
| 665 | **Michinoku** | Japan | winter | 10 | SRR7631424 | yes |
| 694 | **Mansholt** | Netherlands | winter | 10 | SRR7630985 | Yes |
| 697 | **Barcoli** | Netherlands | winter | 9 | SRR7630988 | Yes |
| 705 | **Marana** | Netherlands | winter | 9 | SRR7630980 | Yes |
| 711 | **Bladkool** | Netherlands | winter | 10 | SRR7631375 | No |
| 716 | **Stego** | Netherlands | winter | 9 | SRR7631367 | No |
| 724 | **Giant Rape** | New Zealand | winter | 13 | SRR7631188 | Yes |
| 725 | **Rangi** | New Zealand | winter | 9 | SRR7631099 | Yes |
| 735 | **Niemerczanski 4292** | Poland | winter | 9 | SRR7631331 | Yes |
| 736 | **Pn 3/89** | Poland | winter | 9 | SRR7631330 | Yes |
| 738 | **Rod S- 5125/79** | Poland | winter | 10 | SRR7631434 | Yes |
| 770 | **K 626/72** | Poland | winter | 9 | SRR7631236 | Yes |
| 773 | **K 2040** | Poland | winter | 12 | SRR7631241 | No |
| 781 | **Pur 1** | Poland | winter | 11 | SRR7631389 | Yes |
| 787 | **Boh 585, Boh 5** | Poland | winter | 9 | SRR7631014 | Yes |
| 795 | **Mah 587** | Poland | winter | 9 | SRR7631006 | Yes |
| 819 | **CR 851** | Russian Federation | winter | 11 | SRR7631313 | Yes |
| 937 | **Sv 66/1109** | Sweden | winter | 15 | SRR7631089 | yes |
| 942 | **Sv 7473** | Sweden | winter | 10 | SRR7631204 | Yes |
| 339 | **Santana** | Germany | winter | 9 | SRR7600829 | Yes |
| 703 | **Blako** | Netherlands | winter | 9 | SRR7630986 | Yes |
| 235 | **Liquanta** | Germany | winter | 15 | SRR7600789 | Yes |
| 408 | **Bk 28/78** | Germany | winter | 9 | SRR7600731 | Yes |
| 255 | **Liraglu** | Germany | winter | 16 | SRR7600652 | Yes |
| 27 | **Slapska, Slapy** | Czechoslovakia | winter | 5 | SRR7601056 | No |
| 651 | **CR 2262** | Italy | spring | 9 | SRR7631051 | Yes |
| 336 | **Pura** | Germany | spring | 9 | SRR7601151 | yes |
| 37 | **Ceska Krajowa** | Czechoslovakia | spring | 6 | SRR7600915 | No |
| 63 | **Galant** | USSR | spring | 5 | SRR7600993 | No |
| 70 | **Kruglik** | USSR | spring | 6 | SRR7600994 | No |
| 80 | **Wesway** | Australia | spring | 6 | SRR7600831 | No |
| 88 | **Tribute** | Canada | spring | 5 | SRR7600924 | No |
| 90 | **Oro** | Canada | spring | 5 | SRR7600926 | No |
| 100 | **Altex** | Canada | spring | 6 | SRR7600757 | No |
| 103 | **Target** | Canada | spring | 5 | SRR7600762 | No |
| 108 | **Westar** | Canada | spring | 5 | SRR7600759 | No |
| 113 | **Andor** | Canada | spring | 8 | SRR7600775 | No |
| 122 | **Mozart** | Denmark | spring | 7 | SRR7600782 | No |
| 132 | **Bingo** | Denmark | spring | 5 | SRR7600947 | Yes |
| 146 | **Brio** | France | spring | 9 | SRR7601147 | Yes |
| 175 | **Orpal, Crop** | France | spring | 15 | SRR7600954 | No |
| 189 | **Erglu** | Germany | spring | 5 | SRR7601085 | No |
| 190 | **Adamo** | Germany | spring | 6 | SRR7601084 | Yes |
| 250 | **Korinth** | Germany | spring | 6 | SRR7600785 | Yes |
| 303 | **Aurora** | Germany | spring | 5 | SRR7601118 | No |
| 335 | **Mali** | Germany | spring | 9 | SRR7600827 | No |
| 336 | **Pura** | Germany | spring | 9 | SRR7601151 | No |
| 348 | **Futura** | Germany | spring | 6 | SRR7600930 | No |
| 377 | **Loras** | Germany | spring | 6 | SRR7600970 | No |
| 430 | **Lisandra** | Germany | spring | 5 | SRR7600906 | No |
| 447 | **Liraspa** | Germany | spring | 7 | SRR7601040 | No |
| 460 | **Lirawell** | Germany | spring | 5 | SRR7600679 | No |
| 496 | **Liho** | Germany | spring | 5 | SRR7600699 | No |
| 529 | **Gisora** | Germany | spring | 6 | SRR7601035 | No |
| 707 | **Hankkija's Lauri** | Netherlands | spring | 6 | SRR7631371 | No |
| 764 | **Zachodni** | Poland | spring | 12 | SRR7631070 | No |
| 895 | **Korall** | Sweden | spring | 6 | SRR7631299 | No |
| 903 | **Niklas (svaloef)** | Sweden | spring | 6 | SRR7631214 | No |
| 913 | **Gulliver** | Sweden | spring | 8 | SRR7631036 | Yes |
| 927 | **Omega** | Sweden | spring | 13 | SRR7631442 | No |
| 962 | **Swabi-B11** | Pakistan | spring | 12 | SRR7631407 | No |
| 965 | **Islamsbad** | Pakistan | spring | 9 | SRR7631406 | No |
| 981 | **Chamkani** | Pakistan | spring | 9 | SRR7631132 | No |
| 1093 | **CR 2264** | China | spring | 23 | SRR7631402 | No |

Supplementary Table 3: List of accessions from ERANET ASSYST *B. napus* diversity set tested by PCR for the presence or absence of the insertion

| Accession | Assyst number | Growth type | Insertion |
| --- | --- | --- | --- |
| WESTAR DH | BnASSYST-240 | Spring | No |
| NIKLAS | BnASSYST-253 | Spring | No |
| TARGET | BnASSYST-254 | Spring | No |
| ERGLU | BnASSYST-263 | Spring | No |
| Liho | BnASSYST-271 | Spring | No |
| Marnoo | BnASSYST-278 | Spring | No |
| Olga | BnASSYST-280 | Spring | No |
| Tribute | BnASSYST-284 | Spring | No |
| Wesway | BnASSYST-285 | Spring | No |
| Oro | BnASSYST-287 | Spring | No |
| Mozart | BnASSYST-302 | Spring | No |
| Adamo | BnASSYST-312 | Spring | Yes |
| Altex | BnASSYST-313 | Spring | No |
| Andor | BnASSYST-314 | Spring | No |
| Bingo | BnASSYST-317 | Spring | Yes |
| Erake | BnASSYST-325 | Spring | No |
| Futura | BnASSYST-327 | Spring | No |
| Galant | BnASSYST-328 | Spring | No |
| Giant Xr707 | BnASSYST-329 | Spring | No |
| Gisora | BnASSYST-330 | Spring | No |
| Granit | BnASSYST-333 | Spring | No |
| Gulliver | BnASSYST-334 | Spring | Yes |
| Hankkija's Lauri | BnASSYST-335 | Spring | No |
| Korall | BnASSYST-339 | Spring | No |
| Korinth | BnASSYST-340 | Spring | No |
| Kosa | BnASSYST-341 | Spring | No |
| Kruglik | BnASSYST-342 | Spring | No |
| Lirafox | BnASSYST-344 | Spring | No |
| Lirasol | BnASSYST-345 | Spring | No |
| Liraspa | BnASSYST-346 | Spring | No |
| RAFAL DH1 | BnASSYST-098 | Winter | Yes |
| Lirabon | BnASSYST-119 | Winter | No |
| Leopard | BnASSYST-128 | Winter | Yes |
| Matador | BnASSYST-160 | Winter | Yes |
| Panter | BnASSYST-166 | Winter | Yes |
| CANARD | BnASSYST-185 | Winter | No |
| Silona | BnASSYST-201 | Winter | No |
| SLAPSKA, SLAPY | BnASSYST-212 | Winter | No |
| Dwarf Essex | BnASSYST-193 | Winter | Yes |
| Express617 | BnASSYST-078 | Winter | Yes |
| Nunsdale | BnASSYST-197 | Winter | Yes |
| Matador | BnASSYST-160 | Winter | Yes |
| Savannah | BnASSYST-021 | Winter | Yes |
| Kromerska | BnASSYST-150 | Winter | Yes |
| Rodeo | BnASSYST-014 | Winter | No |
| Rapid | BnASSYST-015 | Winter | Yes |
| Ladoga | BnASSYST-024 | Winter | Yes |
| Capitol | BnASSYST-028 | Winter | Yes |
| Tenor | BnASSYST-054 | Winter | Yes |
| Cooper | BnASSYST-026 | Winter | Yes |
| Samourai | BnASSYST-113 | Winter | Yes |
| Lipid | BnASSYST-034 | Winter | Yes |
| Smart | BnASSYST-046 | Winter | Yes |
| SWGospel | BnASSYST-052 | Winter | Yes |
| Tenor | BnASSYST-054 | Winter | Yes |
| SW Sinatra | BnASSYST-059 | Winter | Yes |
| Nugget | BnASSYST-067 | Winter | Yes |
| Maplus | BnASSYST-117 | Winter | Yes |
| AMBER COMMANCHE DH LINE | BnASSYST-089 | Winter | Yes |
| GROENE GRONINGER SNIJMOES | BnASSYST-218 | Winter | Yes |

Supplementary Table 4: Results of local blast of the insertion sequence upstream of *Bna.SOC1.A05* against Express617 genome

| **Chromosome** | **Start** | **End** | **Identity** | **E-value** |
| --- | --- | --- | --- | --- |
| A05 | 2634532 | 2635132 | 100 | 0.0 |
| A04 | 2135909 | 2136509 | 99.834 | 0.0 |
| A06 | 20055057 | 20054459 | 97.496 | 0.0 |
| A09-random | 305568 | 306178 | 95.581 | 0.0 |

Supplementary Table 5: Results of local blast of the insertion sequence upstream of *Bna.SOC1.A05* against *B. rapa* Z1 genome

| **Chromosome** | **Start** | **End** | **Identity** | **E-value** |
| --- | --- | --- | --- | --- |
| A06 | 49417216 | 49416618 | 97.496 | 0.0 |
| A06 | 49315549 | 49316161 | 95.269 | 0.0 |
| A10 | 9637785 | 9638281 | 93.964 | 0.0 |
| A09 | 43559843 | 43559539 | 83.654 | 9.41e-75 |
